# Exploring the Effect of Annealing on PLA/Carbon Nanotube Nanocomposites: In Search of Efficient PLA/MWCNT Nanocomposites for Electromagnetic Shielding

**DOI:** 10.3390/polym17020246

**Published:** 2025-01-20

**Authors:** Flávio Urbano da Silva, Carlos Bruno Barreto Luna, Fabiano Santana da Silva, José Vinícius Melo Barreto, Debora Pereira Schmitz, Bluma Guenther Soares, Renate Maria Ramos Wellen, Edcleide Maria Araújo

**Affiliations:** 1 Academic Unit of Materials Engineering, Federal University of Campina Grande, Av. Aprígio Veloso, 882-Bodocongó, Campina Grande 58429-900, PB, Brazil; flavio.urbano@ifrn.edu.br (F.U.d.S.);; 2 Federal Institute of Education, Ciência e Tecnologia do Rio Grande do Norte, Natal 59015-000, RN, Brazil; 3 Department of Materials Engineering, Federal University of Paraíba, Cidade Universitária, João Pessoa 58051-900, PB, Brazil; 4 Department of Metallurgical and Materials Engineering—COPPE, PEMM-COPPE, Federal University of Rio de Janeiro, Rio de Janeiro 21941-972, RJ, Brazil

**Keywords:** poly(lactic acid), carbon nanotubes, nanocomposites, extruder processing, electromagnetic shielding

## Abstract

In this research, poly(lactic acid) (PLA) nanocomposites with multi-walled carbon nanotubes (MWCNT) were produced by extrusion, injection, and compression molding, focusing on electromagnetic shielding. Various amounts of carbon nanotubes (MWCNTs) were tested in PLA matrix, specifically ranging from 1 to 4 parts per hundred resin (phr). The resulting nanocomposites were analyzed before and after undergoing annealing heat treatment. It was observed that as the MWCNT content increased, the melt flow index of PLA decreased. This reduction indicates that the nanotubes were effectively accommodated into the PLA chain. The PLA/MWCNT (2 phr) formulation presented the greatest balance of properties, with potential for electromagnetic shielding application. Scanning electron microscopy (SEM) demonstrated that incorporating 2 phr of carbon nanotubes in PLA promoted good distribution, favoring high electrical conductivity and electromagnetic shielding between 20–22 dB (8.2–18 GHz), corresponding to approximately 99% attenuation. Furthermore, its properties, such as elastic modulus (3156 MPa), tensile strength (65.1 MPa), hardness (77.8 Shore D), and heat deflection temperature (55.3 °C), increased compared to pure PLA. After annealing, the PLA/MWCNT (2 phr) nanocomposite underwent a molecular reordering, resulting in an increased crystalline fraction, as confirmed by X-ray diffraction (XRD). However, the electrical conductivity maintained the same order of magnitude, while the electromagnetic shielding varied from 19.7 to 20 dB. The results indicate that these nanocomposites are promising for electromagnetic shielding applications and can be manufactured in the molten state.

## 1. Introduction

In recent years, several studies have been conducted to develop new high-performance polymeric materials through nanotechnology [1,2]. A vital area of this advancement is polymeric nanocomposites, in which the matrix is a polymer, and the dispersed phase has nanometric dimensions [3,4]. Polymeric nanocomposites are a strategy to obtain materials with improved properties using low concentrations of nanofillers (generally between 0.5% and 5% by weight) [5,6]. Depending on the application, polymeric nanocomposites can be developed to meet specific needs, given their great technological versatility for sectors such as the automotive, electronics, biomedical, and packaging industries [7,8]. The use of biopolymers in producing nanocomposites has been widely discussed, as well as the development of more environmentally friendly materials suitable for modern engineering demands [9,10,11]. In this context, poly(lactic acid) (PLA) has emerged as a promising polymer matrix for the manufacture of nanocomposites [12,13,14].

PLA is a promising alternative for reducing dependence on petroleum-based plastics, contributing to a more sustainable future [15]. It is synthesized through the polymerization of lactic acid, which can be derived from the fermentation of carbohydrates such as corn or sugarcane [16]. PLA naturally degrades in environmental conditions when exposed to suitable factors [17]. In terms of mechanical properties, PLA exhibits good rigidity and high tensile strength, with performance comparable to that of crystalline polystyrene (PS) and poly(ethylene terephthalate) (PET) [18]. Moreover, PLA can be processed using various techniques, including injection molding, extrusion, and 3D printing, enabling its application in diverse shapes and products [19]. To further expand its range of applications, PLA has been modified through several approaches, such as producing polymer blends [20,21,22,23], biocomposites reinforced with natural fibers [24,25], crosslinking [26,27], and annealing heat treatment [28,29], all aimed at enhancing its mechanical, thermal, and thermomechanical properties. However, PLA has insulating properties, which limits its use in the electrical sector [30]. Thus, PLA nanocomposites with carbon nanotubes, graphene, and carbon black are being developed to confer multifunctional properties, especially electrical conductivity [31,32,33,34]. Carbon nanotubes (CNT) offer exceptional electrical, thermal, and chemical properties, making them ideal for polymer nanocomposites [35]. The reduced cost of multi-walled carbon nanotubes (MWCNT) has enabled their broader use, enhancing the electrical and electromagnetic properties of insulating polymers [36,37]. Research on PLA nanocomposites with CNTs has increased, focusing on improving phase interactions to optimize material performance [38,39].

Wang et al. [40] developed PLA/CNT mixtures using a two-step process to enhance electrical conductivity and electromagnetic shielding. The method involved preparing CNTs via Pickering emulsion, followed by masterbatch development and melt-processing to form a conductive network. Adding 5.6% CNT achieved 72.2 S/m conductivity, 31.1 dB electromagnetic shielding, and tensile strength above 71.4 MPa. These results highlight the importance of effective CNT distribution and dispersion in the two-step process compared to conventional methods. Younus et al. [41] developed PLA/CNT films (0.5–5% by weight) to enhance mechanical, thermal, and degradation properties. XRD analysis indicated a homogeneous CNT distribution in the PLA matrix. CNTs improved thermal stability, particularly at 5% concentration, while 3% MWCNT increased elongation at break (51.8%) but reduced tensile strength (~64 MPa). The 3% content also provided good stability against UVA radiation. Rivera et al. [42] investigated the potential of lignin as a dispersing agent for carbon nanotubes in the PLA matrix. Adding 5 wt% carbon nanotubes to PLA generated electrical percolation with an electrical conductivity of 2.8 × 10^−7^ S∙cm^−1^. Lignin performed remarkably well dispersing carbon nanotubes in PLA, generating an electrical conductivity of 1.4 × 10^−1^ S∙cm^−1^ for 5 and 1 wt% carbon nanotubes and lignin, respectively. Taweel and Fathy [43] studied the impact of polycaprolactone (PCL) and carbon nanotubes (MWCNT) on PLA properties. CNTs improved the compatibility of the PLA/PCL mixture, enhancing tensile properties. They also accelerated PLA crystallization through a nucleating effect and increased thermal stability, compared to the PLA/PCL mixture. Kaczor et al. [44] studied the effect of reprocessing on PLA/CNT mixtures. Differential scanning calorimetry (DSC) analysis showed that cold crystallization temperature decreased, while enthalpy increased after each reprocessing cycle. PLA degradation during extrusion increased the melt flow index, with more intense degradation in PLA/MWCNT mixtures, indicating that carbon nanotubes may have a catalytic effect, accelerating the chain scission process.

Recently [45,46], the annealing process of PLA reinforced with carbon-based nanofillers has been explored, aiming to alleviate molding stresses and improve crystallinity and compatibility with the dispersed phase. During annealing, the polymer chains reorganize, increasing crystallinity. This may favor greater contact between the conductive nanofillers at the spherulites contours, which probably favors the electrical conduction process. In addition, there is a tendency for carbon nanotubes to agglomerate, which requires efficient processing to increase homogenization in the PLA matrix. The good distribution and dispersion of carbon nanotubes in the extruder can contribute to the manufacture of polymer nanocomposites with balanced properties, such as mechanical and electrical. Given this, the screw profile of the extruder can lead to greater effectiveness in PLA/carbon nanotube nanocomposites, such as reducing agglomerates and increasing the contact area between the components, which undoubtedly improves the properties. Few reports in the scientific literature evaluate the joint influence of using an extruder with distributive and dispersive mixing elements, in addition to post-molding heat treatment, aiming for an efficient distribution of nanotubes during processing in the molten state. This constitutes a differentiated procedural approach, which contributed to evaluating the final impact on properties and morphology.

Therefore, the present research aimed to develop PLA nanocomposites with different contents of multi-walled carbon nanotubes (MWCNTs) to obtain an efficient material for electromagnetic shielding. Additionally, this study evaluated the impact of annealing treatment to identify the optimal composition that enhances electrical and electromagnetic properties, aiming at the production of efficient materials to attenuate electromagnetic radiation.

## 2. Materials and Methods

### 2.1. Materials

PLA was used as the polymer matrix, identified by the code 3D Lab, provided by NatureWorks. The specifications for PLA include a density of 1.24 g/cm³ (ASTM D792), a relative viscosity of 4.0 (ASTM D5225), and a melt flow index ranging from 7 to 9 g/10 min (ASTM D1238). Its glass transition temperature (T_g_) is between 55 and 60 °C (ASTM D3418), with a molecular weight of 11152 kDa [47]. Multi-walled carbon nanotubes (MWCNT), utilized as conductive fillers, have a purity greater than 99%. These nanotubes are supplied in the form of black powder and are produced by the CVD method at Yuechuang Technology. Their characteristics include an outer diameter of 8–15 nm, a length of 10–50 μm, a specific surface area of over 230 m^2^/g, an electrical resistivity of 1412 μΩ m, and an ash content of less than 0.1%.

### 2.2. Extruder Processing

**Figure 1:**
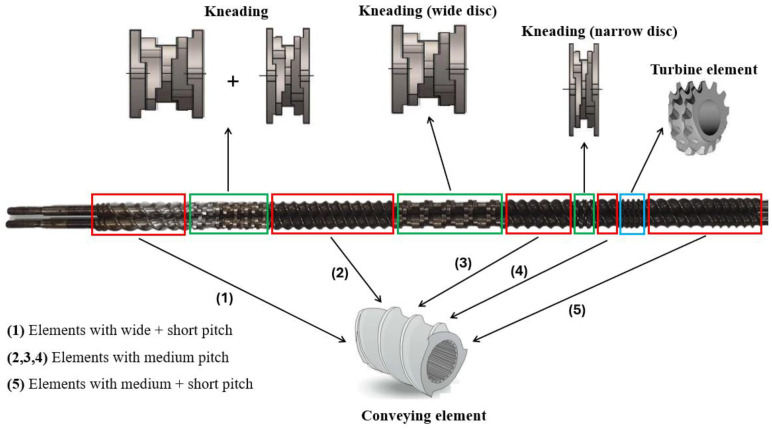
Screw configuration used in the extruder, adapted from the literature [48,49]. Red: transport elements; Green: kneading elements; Blue: turbine elements.

Pure PLA and carbon nanotubes (MWCNTs) were dried to eliminate moisture in a vacuum oven (Ethiktechnology, model 440-1D) at 60 °C for 24 h. Subsequently, the PLA/MWCNT concentrates were prepared in a torque rheometer (Thermoscientific Polylab QC, Waltham, MA, USA) with operating parameters of 180 °C and rotor speed of 60 rpm for 3 min. The concentrates were ground in a knife mill (WEG, model CPS-9) to obtain flakes for dilution in the extruder. The adopted compositions were PLA/MWCNT (% by mass/parts per hundred of resin (phr), in the proportions of 100/1, 100/2, 100/3, and 100/4. Carbon nanotubes were used as a complementary additive to promote electrical conduction. PLA/MWCNT nanocomposites were processed in a modular co-rotational twin-screw extruder, model ZSK (D = 18 mm and L/D = 40), from Coperion Werner-Pfleiderer (Stuttgart-Feuerbach, Germany). In addition, pure PLA was obtained under the same experimental conditions for comparative purposes. The screw in the extruder consists of distributive and dispersive elements, as shown in Figure 1. The screw configuration is suitable for high shear rates to maximize the distribution and dispersion of nanofillers. The following parameters were used during processing:

- Temperature profile: 160 °C–170 °C–170 °C–175 °C–180 °C–180 °C–190 °C;
- Screw rotation speed: 250 rpm;
- Feed rate in extruder: 3 kg/h.

After the extrusion process, the pure PLA and PLA/MWCNT nanocomposites were granulated, dried (60 °C/24 h), and molded by injection and compression.

### 2.3. Injection and Compression Molding

The extruded materials were manufactured by injection molding into samples for impact (ASTM D256), tensile (ASTM D638), Shore D hardness (ASTM D2240), and HDT (ASTM D648) testing using an Arburg injection molding machine (model Allrounder 207C Golden Edition, Loßburg, Germany). Injection molding was performed according to the following parameters:

- Temperature profile: 160 °C–170 °C–175 °C–180 °C–190 °C;
- Mold temperature: 20 °C;
- Cooling time: 25 s;
- Injection pressure: 1200 bar;
- Holding pressure: 800 bar.

Compression molding was performed on extruded PLA pellets and PLA/MWCNT nanocomposites, aiming at the production of plates (length of 8 cm, width of 8 cm, and thickness of 0.2 cm) for electrical conductivity and electromagnetic shielding tests. The plates were manufactured at a temperature of 190 °C, following the following thermal cycles:

- Preload of 2 tons for 2 min;
- Final load of 8 tons for 3 min;
- Cooling at room temperature for 10 min, under a load of 50 N.

### 2.4. Annealing Heat Treatment

The injection and compression molded samples were subjected to annealing heat treatment in a vacuum oven (Ethiktechnology, model 440-1D, São Paulo, Brazil), at 90 °C, under a pressure of 500 mmHg for 5 h [50]. The nomenclature adopted will be PLAT, where “T” refers to the annealing heat treatment.

### 2.5. Nanocomposite Characterization

The melt flow index (MFI) was determined using a Hebert Lambert plastometer (CLP Schneider 3210, São Paulo, Brazil). The analysis followed the ASTM D1238 standard, with conditions of 210 °C and 2.16 kg. The test was performed on extruded PLA samples and PLA/MWCNT nanocomposites, and the results were analyzed based on the average of ten samples.

X-ray diffraction (XRD) was conducted on a Bruker equipment (model D2 PHASER, Billerica, MA, USA), using Kα radiation from copper (Cu), in the range of 5–30°, voltage/current of 30 kV/10 mA, and step of 0.02°. The degree of crystallinity (X_c_) was calculated using Equation (1):

(1) $$X_{c}=\frac{A_{c}}{A_{c}+A_{a}}$$

where A_c_ = crystalline fraction and A_a_ = amorphous fraction. The areas of the crystalline and amorphous fraction were determined through integration, using the Origin software (Version 8.5).

Fourier transform infrared spectroscopy (FTIR) analysis was conducted using the attenuated total reflectance (ATR) method on a Bruker Alpha II Spectrometer (Billerica, MA, USA). The tests were performed on the surface of injected samples with a thickness of 3.2 mm. Scanning was carried out from 4000 to 400 cm^−1^, with a resolution of 4 cm^−1^ and a total of 32 scans.

The electrical conductivity (σ) test was performed on compression molded films with a thickness of less than 1 mm. Measurements were performed with a Keithley electrometer (model 8009, Cleveland, OH, USA) and the volumetric method. The parameters adopted were a current of 20 mA and a voltage of 1 volt for 2 min.

The electromagnetic shielding (EMI SE) test was performed on an Agilent Technologies vector network analyzer (VNA) (Santa Clara, California), model N5230C PNA-L. The equipment is coupled to a waveguide corresponding to the X-band (8.2–12.4 GHz) and Ku-band (12.4–18 GHz) frequency ranges. The samples with approximate thickness of 2 mm were compression molded.

The Izod impact strength was determined on notched test specimens using a Ceast Resil 5.5 instrument equipped with a 2.75 J pendulum (Norwood, MA, USA), following ASTM D256. The tests were conducted at room temperature, and the results were averaged over ten samples.

The tensile strength was evaluated using an Oswaldo Filizola BME universal testing machine (São Paulo, Brazil), in accordance with the ASTM D638 standard. The test was conducted at room temperature with a deformation speed of 5 mm/min and a 20 kN load cell. The results were analyzed based on the average of ten samples.

The Shore D hardness test was conducted using Metrotokyo equipment (São Paulo, Brazil), following the guidelines of ASTM D2240. A 50 N load was applied to the sample at ten random points, with the indenter held in place for 10 s.

The heat deflection temperature (HDT) analysis was performed using CEAST equipment (Norwood, MA, USA), model HDT 6 VICAT, in accordance with ASTM D648 recommendations. The heating rate was set to 120 °C/h under a load of 1.82 MPa. Silicone oil was used as the immersion medium, and the result was recorded when a deflection of 0.25 mm was observed in the sample. The analysis was based on the average of three samples.

Differential scanning calorimetry (DSC) was performed on Shimadzu equipment (DSC-60Plus, Kyoto, Japan), using nitrogen as a carrier gas (50 mL/min). The scan was 30 to 200 °C (heating–cooling–heating), with a heating rate of 10 °C/min, 2 min isotherm, and mass of 3 mg. The degree of crystallinity (X_c_) of PLA was calculated using Equation (2):

(2) $$X_{c}=\frac{{∆H}_{m}-{∆H}_{cc}}{{∆H}_{m100\%}}\;*100\%$$

where ∆H_m_= crystalline melting enthalpy obtained by DSC; ∆H_cc_ = cold crystallization enthalpy; ∆H_m100%_ = melting enthalpy of PLA with 100% crystallinity, 93.7 J/g.

Scanning electron microscopy (SEM) was performed on the fractured surfaces of the samples using a TESCAN VEGA 4 microscope (Brno, Czech Republic), under high vacuum and an accelerating voltage of 5 keV. Pure PLA and PLA/MWCNT nanocomposites were gold-coated for 3 min.

## 3. Results and Discussion

### 3.1. Melt Flow Index (MFI)

**Figure 2:**
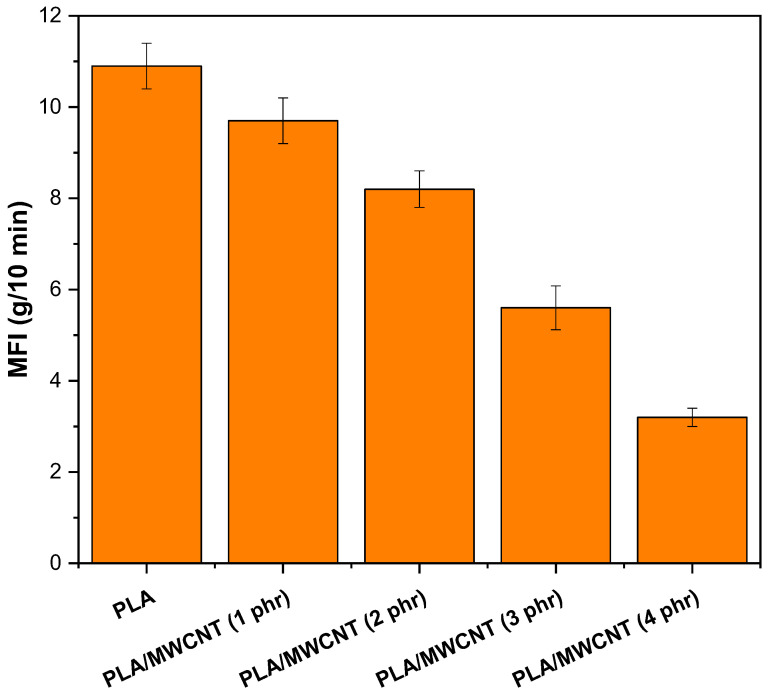
MFI results for pure PLA and PLA/MWCNT nanocomposites.

The melt flow index (MFI) is widely used in the industry to assess the flow behavior of polymers due to the simplicity and agility of the analysis using a plastometer. Figure 2 shows the MFI results for pure PLA and PLA/MWCNT nanocomposites with varying MWCNT contents.

Pure PLA exhibited an MFI of 10.9 g/10 min after extrusion processing, which is higher than the manufacturer’s reported range of 7–9 g/10 min. A similar result for PLA (Ingeo™ 3D850) was reported by Wang et al. [51], with an MFI of 11.1 g/10 min (210 °C/2.16 kg). The literature [52,53] indicates that PLA is sensitive to thermomechanical processing, which can result in chain scission and reduced viscosity. Incorporating 1 phr of MWCNT into the PLA matrix slightly reduced the MFI to 9.7 g/10 min, indicating increased viscosity. As the MWCNT content increased to 2, 3, and 4 phr, a progressive reduction in MFI was observed, corresponding to a further increase in viscosity. This suggests higher yield strength in the PLA/MWCNT nanocomposites compared to neat PLA. Carbon nanotubes distributed along the PLA chains restricted molecular flow, contributing to increased viscosity and reduced MFI. These findings align with the literature [54,55], which suggests that polymer-MWCNT and MWCNT-MWCNT interactions are responsible for the observed decline in MFI.

### 3.2. Fourier Transform Infrared Spectroscopy (FTIR)

**Figure 3:**
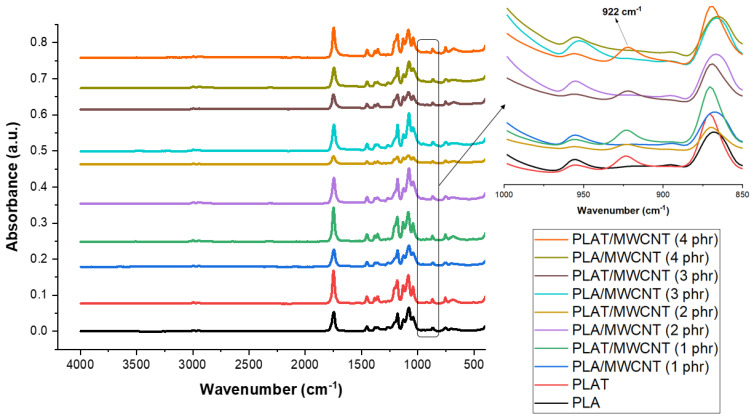
FTIR curves for pure PLA and PLA/MWCT nanocomposites, with and without annealing treatment. Normalization of FTIR spectra was performed using Bruker OPUS software (Version 9.0).

Figure 3 presents the FTIR spectra of pure PLA and PLA/MWCNT nanocomposites with varying carbon nanotube contents and annealing treatment.

The main bands observed in PLA are 2946 cm^−1^ and 2995 cm^−1^ (asymmetric and symmetric stretching of the C-H bond in the CH_3_ group); 1752 cm^−1^ stretching of the carbonyl group (C = O); 1450 cm^−1^ and 1355 cm^−1^ (asymmetric deformation of the C-H bond in the methyl group (CH_3_)); and 1267 cm^−1^, 1178 cm^−1^, and 1086 cm^−1^ (stretching of the C-O bonds in the C-O-C group) [56,57]. The incorporation of carbon nanotubes into the PLA matrix did not alter the main absorption bands; they were maintained. The annealing process in PLA and PLA/MWCNT nanocomposites can induce changes in the molecular and structural order, which may modify the FTIR spectra [20]. Annealing promotes the reorganization of the PLA chains, which can enhance crystallinity. This can be assessed by FTIR, as increased crystallinity in PLA is associated with the emergence of bands around 921 cm^−1^ and 1208 cm^−1^, as reported in the literature [58]. As shown in Figure 3 (magnification 850–1000 cm^−1^), both pure PLAT and the PLAT/MWCNT nanocomposites exhibited a new band at 922 cm^−1^, suggesting that annealing resulted in materials with higher crystallinity, which is supported by the XRD results presented below. This is significant for the final performance of the product, as effective annealing in PLA can alter its mechanical, thermomechanical, and electrical conductivity properties.

### 3.3. X-Ray Diffraction (XRD)

**Figure 4:**
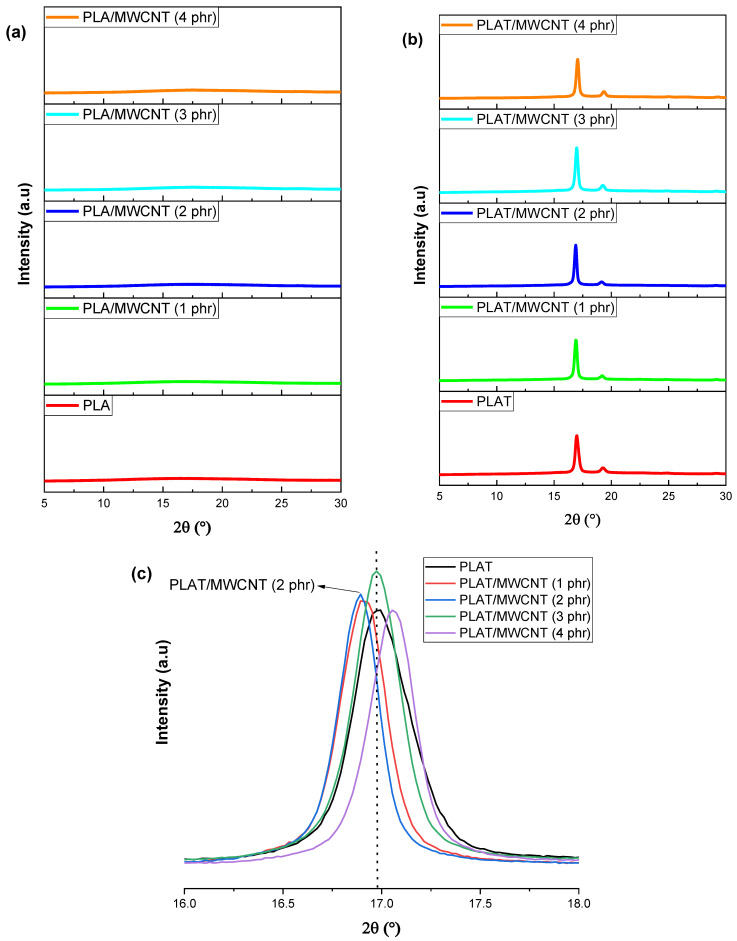
XRD results for pure PLA and PLA/MWCNT nanocomposites, with different MWCNT contents, for (**a**) without annealing; (**b**) with annealing; (**c**) magnification from 16 to 18°.

The annealing process in PLA promotes the formation of a more ordered structure in the polymer chains by promoting reorganization in a more crystalline fraction [59]. During annealing between T_g_ and T_m_, molecular mobility increased, facilitating the enhancement of the crystalline lamellae. Figure 4a,b show the XRD diffractograms for pure PLA and PLA/MWCNT nanocomposites, both with and without annealing heat treatment.

Figure 4a shows that both pure PLA and PLA/MWCNT nanocomposites, regardless of the carbon nanotube content, exhibited behavior characteristic of an amorphous material, with no crystalline peaks. Incorporating carbon nanotubes into the PLA matrix was insufficient to induce molecular organization, maintaining the predominantly amorphous behavior. This confirms the result obtained by DSC later. In contrast, crystalline peaks are observed in Figure 4b when PLAT and PLAT/MWCNT nanocomposites were subjected to annealing. This suggests that annealing promoted molecular organization, resulting in more crystalline materials. After heat treatment, well-defined and close peaks were observed around 2θ = 17° and 2θ = 19.4°, corresponding to the (110)/(200) and (203) planes, respectively [60]. According to the literature [61], these peaks are characteristic of the α phase of PLA. For the PLAT/MWCNT nanocomposites with 1 phr and 2 phr, the 2θ values for the (110)/(200) reflection showed a slight shift to smaller values (see Figure 4c). Consequently, the d-spacing in the crystal structures increased to 1 and 2 phr of MWCNT (see Table 1), suggesting the incorporation of carbon nanotubes into the PLAT chains.

Table 1 shows the degree of crystallinity (X_c_), basal spacing (d), and crystal size (L), obtained by XRD of pure PLAT and PLAT/MWACNT nanocomposites, after annealing treatment.

The PLAT exhibited a degree of crystallinity of approximately 46%, a value similar to that reported in the literature [62] after annealing. Adding 1 phr of MWCNT to the PLAT matrix increased the degree of crystallinity to 53.9%, indicating a nucleating effect of the carbon nanotubes. When the MWCNT concentration was increased to 2 phr, a significant rise in crystallinity was observed, reaching 63.7%. This suggests a good distribution of carbon nanotubes in the PLAT at 2 phr, which enhanced the nucleation process and promoted the formation of stable crystals. At a concentration of 2 phr of MWCNT, there was good accommodation of the carbon nanotubes into the PLAT chains, which helped reduce the critical energy required to consolidate crystalline nuclei. However, at concentrations of 3 phr and 4 phr of MWCNT, the degree of crystallinity decreased to 51% and 47.2%, respectively, compared to PLAT/MWCNT (2 phr). Thus, 2 phr MWCNT in PLAT indicated an ideal concentration to optimize the degree of crystallinity, while concentrations higher than this value negatively affected crystallinity. In other words, the 3 phr and 4 phr MWCNT concentrations hindered the formation of stable nuclei for crystal growth and consolidation, leading to a decrease in crystallinity compared to the system with 2 phr of MWCNT.

### 3.4. Scanning Electron Microscopy (SEM)

**Figure 5:**
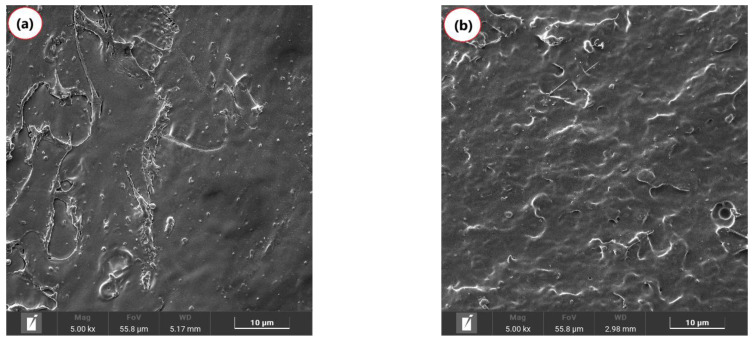
Morphology obtained by SEM with 5000× magnification, for (**a**) PLA; (**b**) PLAT under the effect of heat treatment.

Figure 5 presents the morphology observed by SEM for PLA before and after annealing heat treatment. In Figure 5a, a brittle fracture surface was observed, showing little plastic deformation, consistent with findings reported in the literature for PLA [63]. After the annealing, PLAT exhibited a more homogeneous surface. However, it retained its brittle fracture behavior without significantly increasing ductility.

**Figure 6:**
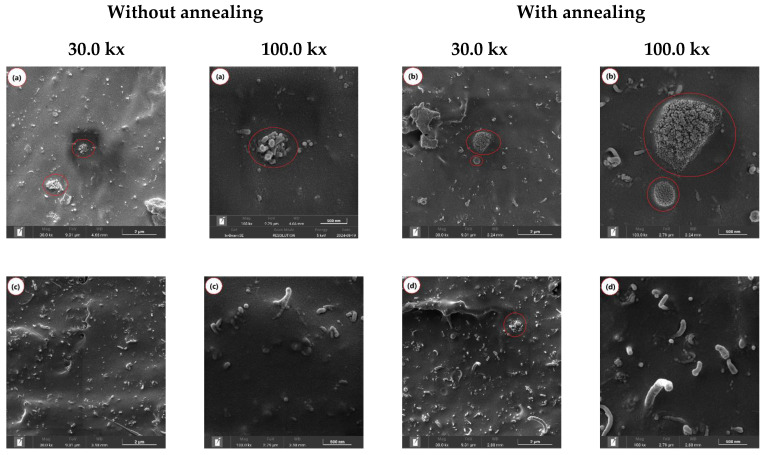

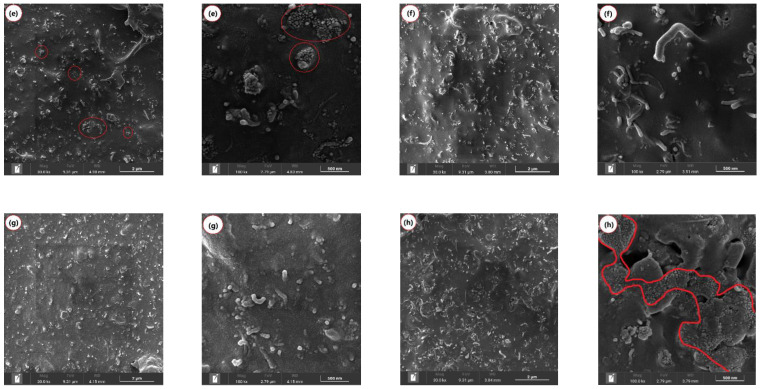
Evolution of the morphology of nanocomposites with different carbon nanotube contents, under the effect of annealing, for (**a**) PLA/MWCNT (1 phr) and (**b**) PLAT/MWCNT (1 phr); (**c**) PLA/MWCNT (2 phr) and (**d**) PLAT/MWCNT (2 phr); (**e**) PLA/MWCNT (3 phr) and (**f**) PLAT/MWCNT (3 phr); (**g**) PLA/MWCNT (4 phr) and (**h**) PLAT/MWCNT (4 phr).

To investigate the morphology of the PLA/MWCNT and PLAT/MWCNT nanocomposites, SEM images were taken from the fracture surfaces of the impact specimens. Figure 6a–h illustrate the morphological evolution of the nanocomposites both before and after annealing.

In Figure 6a, the PLA/MWCNT (1 phr) nanocomposite exhibited a poor distribution of carbon nanotubes, resulting in regions with high concentrations (red circle) and areas where the particles were spaced too far apart. This led to reduced electrical conductivity, as noted later. As reported in the literature [64], carbon nanotubes tend to agglomerate due to Van der Waals forces and their high surface area. The annealing process of the PLAT/MWCNT (1 phr) nanocomposite, as shown in Figure 6b, did not enhance the distribution of carbon nanotubes the PLA matrix. Instead, it resulted in a greater formation of agglomerates with higher particle concentrations (see red circle), indicating poor distribution. This could negatively affect the electrical conductivity of the PLAT/MWCNT (1 phr) nanocomposite, as it reduces the contact between the carbon nanotubes throughout the PLA matrix [65]. In the 2–4 phr MWCNT range, a noticeable change in the evolution of PLA morphology was observed compared to the 1 phr MWCNT content. A greater distribution of carbon nanotubes was observed in the PLA/MWCNT (2, 3, and 4 phr) nanocomposites, which directly contributes to reducing the interparticle distance between carbon nanotubes and increasing electrical conductivity, as verified in Table 2 below. In Figure 6c, the 2 phr MWCNT content resulted in a good distribution of carbon nanotubes in the PLA matrix. Moreover, no particles were detached from the carbon nanotubes during the impact test, indicating good interfacial wettability. This is important for the material’s tensile strength, as it enhances the reinforcing effect [66]. After applying the annealing process to the PLAT/MWCNT (2 phr) nanocomposite, the good distribution of carbon nanotubes was maintained. The presence of agglomerates (red circle) was also observed, as shown in Figure 6d. Nevertheless, the level of agglomeration was lower compared to the PLA/MWCNT (1 phr) nanocomposite. The PLA/MWCNT (2 phr) and PLAT/MWCNT (2 phr) nanocomposites exhibited carbon nanotubes with fibrous characteristics, suggesting a higher aspect ratio in these samples.

In Figure 6e, the PLA/MWCNT (3 phr) nanocomposite exhibited a morphology with a good distribution of carbon nanotubes but also showed agglomerates with rounded particles (red circle). In contrast, after applying the annealing process, the PLAT/MWCNT (3 phr) nanocomposite displayed a more stable morphology, with an improved distribution of carbon nanotubes and a higher aspect ratio, as shown in Figure 6f. Apparently, for MWCNT concentrations above 2 phr, the annealing process favored a better accommodation and distribution of carbon nanotubes in the PLA matrix. As the MWCNT content increased to 4 phr, the distribution of carbon nanotubes became more intense and uniform, with particles close together, as shown in Figure 6g. A similar morphological trend was observed for the PLA/MWCNT (4 phr) nanocomposite, with a wide distribution of carbon nanotubes. As shown in Figure 6h, the annealing process promoted the formation of an interconnected network of carbon nanotubes (see red highlight), suggesting establishing an electrical percolation path. This morphological behavior was not observed for the PLA/MWCNT (4 phr) nanocomposite, likely due to the predominance of amorphous chains, as indicated by the XRD analysis. After annealing the PLAT/MWCNT (4 phr) nanocomposite, molecular organization occurred, forming more crystalline fractions. This likely caused the carbon nanotubes to shift toward the interspherulitic amorphous regions of the PLA. As a result, a more pronounced formation of the percolation path was observed, as shown in Figure 6h at 100,000× magnification.

**Figure 7:**
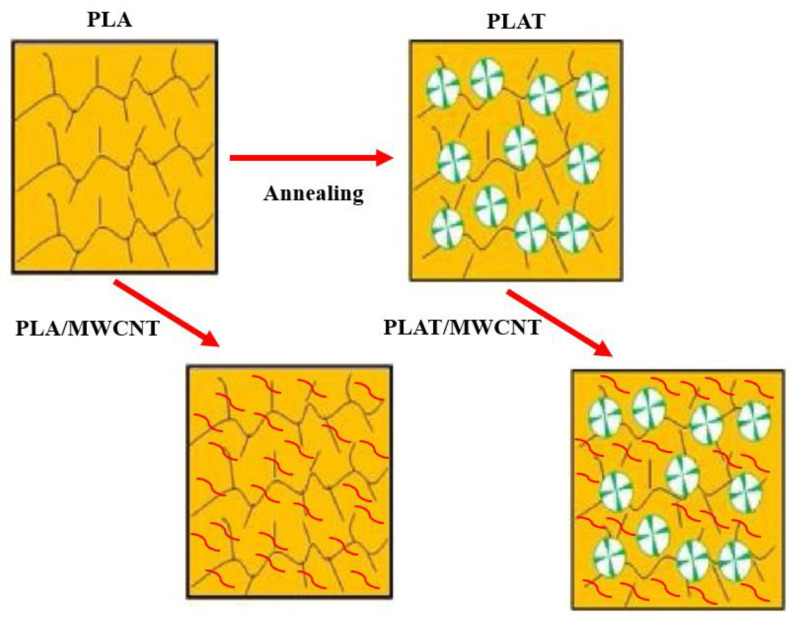
Representation of the morphology of PLA and nanocomposites, with the annealing process. Marking in red represents carbon nanotubes. Adapted from the literature [67] with permission from Elsevier, under license 5905520821879.

Figure 7 illustrates the evolution of the morphology of PLA and the nanocomposites, before and after the annealing process. Annealing increased the crystallinity of PLA, as confirmed by the XRD analysis. During the heating process at 90 °C, the PLA chains gained more freedom to reorganize into ordered structures, forming crystals. In the PLA/MWCNT nanocomposites, the carbon nanotubes were dispersed along the amorphous PLA chains. In contrast, the PLAT/MWCNT nanocomposites exhibited a higher crystalline fraction, being more ordered, cohesive, and dense. This possibly leads to the rejection of the carbon nanotubes by the growing crystals and, therefore, being deposited in amorphous interspherulitic regions (see Figure 6h).

### 3.5. Electrical and Electromagnetic Properties

Table 2 presents the electrical conductivity results for pure PLA and nanocomposites as a function of the MWCNT concentration and the annealing process.

As described in the literature [68], materials can be classified as insulators (10^−11^ to 10^−22^ S/cm), semiconductors (10^−2^ to 10^−9^ S/cm), conductors (10^2^ S/cm), or superconductors (10^20^ S/cm). Pure PLA exhibited an electrical conductivity of 8.13 × 10^−11^ S/cm, classifying it as an insulating material, a behavior consistent with that observed by Silva et al. [69]. With the addition of carbon nanotubes, the electrical conductivity of PLA increased, displaying a typical electrical percolation behavior. A low concentration of 1 phr MWCNT in the PLA matrix raised the electrical conductivity to 2.85 × 10^−7^ S/cm, contributing to the transition from insulation to semiconducting material. The electrical conductivity of the PLA/MWCNT nanocomposites showed a significant increase, by a factor of approximately 10^5^, in the range of 2 to 4 phr of MWCNT. This indicates that the distribution of carbon nanotubes in PLA was effective in producing a significant increase in the electrical conductivity of the nanocomposites, which aligns with the SEM trend. Although the PLA/MWCNT nanocomposites with 3 phr and 4 phr exhibited higher electrical conductivity than the PLA/MWCNT (2 phr), their performance was similar, with conductivity remaining in the same order of magnitude (10^−6^ S/cm). This suggests that 2 phr of MWCNT was sufficient to achieve effective distribution and dispersion in PLA, enabling electrical percolation. This finding indicates that the concentration of 2 phr of MWCNT was sufficient under the experimental conditions in the extruder to optimize the distribution in the PLA matrix, generating electrical conductivity in the order of 10^−6^ S/cm. Therefore, it was not necessary to use higher MWCNT contents (3 and 4 phr), thus contributing to reducing the cost of the final product.

The annealing process in PLAT maintained the insulating behavior with an electrical conductivity of 8.54 × 10^−11^ S/cm, but with a 5% increase in this property. As reported in the literature [70], the electrical conductivity in conductive composites and nanocomposites is affected by the degree of crystallinity of the polymer matrix, given the accommodation of the conductive charge in the amorphous region. The increase in the crystalline fraction causes a reduction in the amorphous phase, thus contributing to greater physical contact between conductive particles, generating an electrical percolation path (see Figure 7). In principle, the PLAT/MWCNT (1 phr) and PLAT/MWCNT (2 phr) nanocomposites suffered a decline in electrical conductivity compared to the analogs without annealing treatment. However, the order of magnitude was maintained at 10^−6^ S/cm. As verified in the XRD, the PLAT/MWCNT (1 phr) and PLAT/MWCNT (2 phr) nanocomposites exhibited the highest values of crystallinity degree, but this did not reflect in the improvement of electrical conductivity. Weidenfeller et al. [71] indicated that simply increasing the degree of crystallinity is not the sole determining factor for improving electrical conductivity; the size and degree of perfection of the crystallites must also be considered. As verified in the XRD, the PLAT/MWCNT (1 phr) and PLAT/MWCNT (2 phr) nanocomposites exhibited less perfect crystals, which probably caused an irregular compaction of carbon nanotubes in the amorphous fraction of PLA. This resulted in some segregation, which impaired electrical conduction. This assumption was verified in the morphology by SEM, as shown in Figure 6b,d. As the MWCNT content increased to 3 phr and 4 phr, a change in electrical conductivity was observed. The PLAT/MWCNT (3 phr) and PLAT/MWCNT (4 phr) nanocomposites increased electrical conductivity by 9.9% and 15.5%, respectively, compared to the PLA/MWCNT (3 phr) and PLA/MWCNT (4 phr) systems. The formation of a more stable crystalline morphology was possibly established in these nanocomposites, contributing to a greater electrical response. Similar behavior was observed by [72,73], suggesting that the formation of larger crystals promotes the development of a selectively distributed structure of conductive charges in the amorphous phase, thereby enhancing electrical conductivity. As shown in the SEM images, the PLAT/MWCNT (3 phr) and PLAT/MWCNT (4 phr) nanocomposites, after annealing treatment, exhibited a good distribution of carbon nanotubes, with the 4 phr MWCNT content even forming an interconnected network. This network structure helps explain the improved electrical conductivity performance.

**Figure 8:**
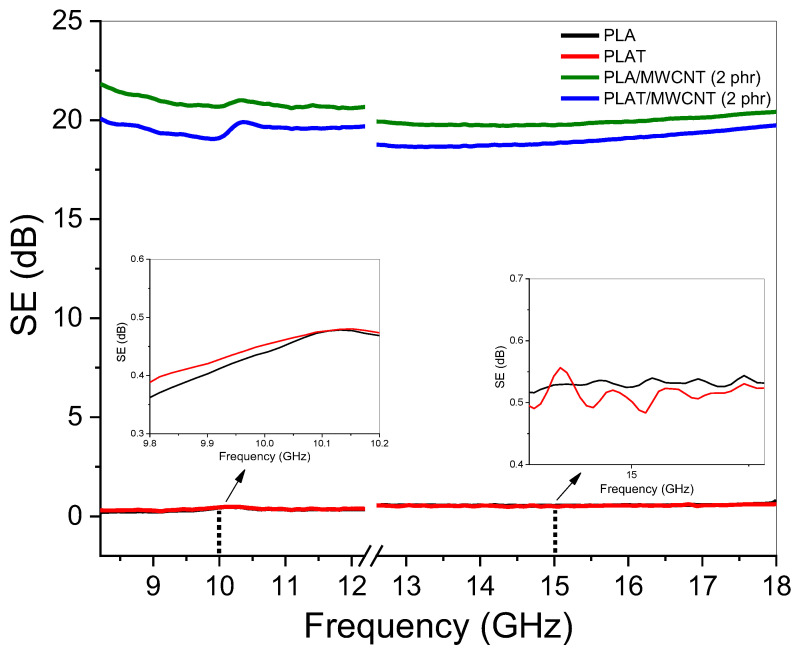
Shielding efficiency (EMI SE) curves for PLA and PLA/MWCNT nanocomposite, before and after annealing.

From an application standpoint, materials with electrical conductivity greater than 10^−8^ S/cm are suitable for electrostatic charge dissipation [74]. For electrostatic painting and protection against electromagnetic interference, conductivities in the range of 10^−6^ S/cm and 10^−1^ S/cm, respectively, are required [75,76]. As shown in Table 2, the electrical conductivity values of the PLA/MWCNT and PLAT/MWCNT nanocomposites are promising for electrostatic dissipation. In the MWCNT concentration range of 2 to 4 phr, the electrical conductivity remained in the order of 10^−6^ S/cm. Based on this, the PLA/MWCNT (2 phr) and PLAT/MWCNT (2 phr) nanocomposites were selected for evaluation of their electromagnetic shielding potential, as using a lower amount of MWCNT helps reduce the cost of the final product. Figure 8 shows the electromagnetic shielding (EMI SE) behavior for PLA and PLA/MWCNT (2 phr) nanocomposite, under annealing effect.

Figure 8 shows that both PLA and PLAT after annealing exhibited little to no attenuation of electromagnetic radiation, as they are insulating materials. However, when only 2 phr of MWCNT was added to the PLA matrix, a shielding efficiency in the range of 20–22 dB was observed across the entire analyzed frequency range, corresponding to approximately 99% attenuation. Carbon nanotubes are used in the fabrication of nanocomposites because of their ability to enhance electrical conductivity, increase interaction with electromagnetic radiation, and improve the electromagnetic shielding effect [77]. As observed in the SEM analysis, the PLA/MWCNT (2 phr) nanocomposite displayed a homogeneous distribution of carbon nanotubes, facilitating the formation of a protective network against electromagnetic radiation, resulting in high attenuation capacity. It appears that the screw configuration in the extruder, featuring both distributive and dispersive mixing elements, contributed to the excellent morphological stability, primarily due to the uniform distribution of carbon nanotubes. The shielding efficiency of the PLA/MWCNT (2 phr) nanocomposite holds practical technological significance, as commercial components designed for attenuation must achieve at least 20 dB [77,78]. When the PLAT/MWCNT (2 phr) nanocomposite was subjected to the annealing treatment, a reduction in the electromagnetic shielding effectiveness was observed, with values ranging from 19.7 to 20 dB, which corroborates the trend of electrical conductivity. MWCNT agglomerates indicate a less efficient distribution in the PLA, which compromised the barrier effect against electromagnetic radiation. However, it is worth noting that the electromagnetic shielding value of the PLAT/MWCNT (2 phr) nanocomposite is still within the viable limit for product development.

**Figure 9:**
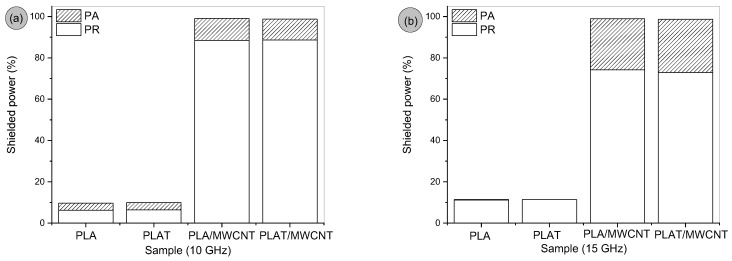
Behavior of the absorption and reflection mechanism of PLA and nanocomposites, for: (**a**) 10 GHz; (**b**) 15 GHz.

The shielded energy can be expressed as reflected energy (PR) and absorbed energy (PA). Figure 9a,b illustrates the absorption and reflection mechanisms contribution for the PLA and the PLA/MWCNT (2 phr) nanocomposite, with and without the annealing process. The graphs below show the shielded energy values at the frequencies of 10 GHz (corresponding to the X band) and 15 GHz (corresponding to the Ku band). In the PLA/MWCNT and PLAT/MWCNT nanocomposites, the predominant shielding mechanism was the reflection of the incident electromagnetic wave. The efficiency of shielding by reflection is directly associated with the electrical conductivity of the material [79]. As indicated by the results of the electrical conductivity test in Table 2, the PLA/MWCNT (2 phr) and PLAT/MWCNT (2 phr) nanocomposites presented conductivity values in the order of 10^−6^ S/cm, which was reflected in similar results in the absorption and reflection mechanisms.

**Figure 10:**
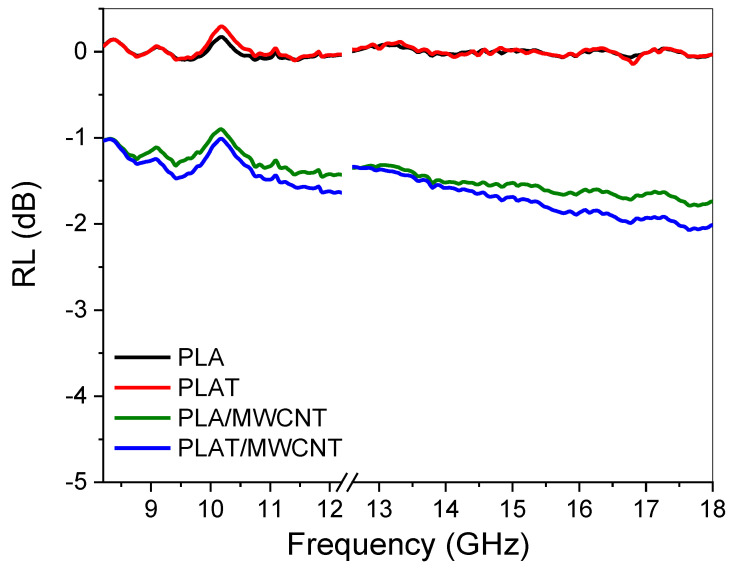
Reflection loss behavior for PLA and nanocomposites.

Figure 10 shows the reflection loss (RL) for PLA and nanocomposites with annealing heat treatment. PLA and PLAT exhibited typical behavior of insulating and transparent materials, with the reflection loss varying around 0 dB. Due to the high electromagnetic shielding (SE) response of the PLA/MWCNT and PLAT/MWCNT nanocomposites, the reflection loss was low, since this loss is directly related to the absorption capacity of the material. As shown in Figure 9, the absorption represented a small fraction of the absorption response of the PLA/MWCNT and PLAT/MWCNT nanocomposites. Thus, the incident wave was almost completely reflected, resulting in a low reflection loss (RL) value.

### 3.6. Mechanical Properties

**Figure 11:**
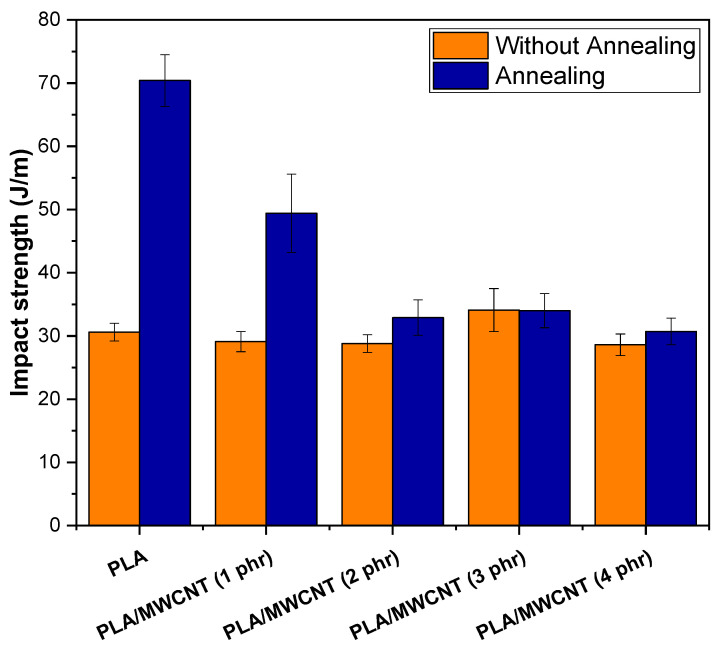
Impact strength of PLA and PLA/MWCNT nanocomposites.

Figure 11 shows the impact strength results of PLA and PLA/MWCNT nanocomposites considering different carbon nanotube contents and the effect of annealing.

The brittle behavior of PLA was evidenced by its low impact strength of 30.6 J/m, a value consistent with data found in the literature [80,81]. Incorporating MWCNT into the PLA matrix led to a slight reduction in impact strength, ranging from 28.6 to 29.1 J/m for the 1 phr, 2 phr, and 4 phr MWCNT contents, compared to pure PLA. The only exception was the PLA/MWCNT (3 phr) nanocomposite, which exhibited a slight increase in impact strength. However, considering the experimental error margin, the impact strength values for the PLA/MWCNT nanocomposites and pure PLA were similar. Thus, PLA was modified with low concentrations of MWCNT to achieve multifunctional properties, particularly maintaining impact strength while acquiring electrical conductivity.

Annealing of PLAT resulted in a significant increase in impact strength, reaching 70.4 J/m, representing a 130% improvement over neat PLA. This suggests that the crystallization of PLAT enhanced its toughness, possibly through a mechanism in which the crystals helped to retard crack propagation [82], thus preventing premature failure as observed in unannealed PLA. The effect of annealing on increasing the impact strength of PLA has also been reported in the literature [83,84], although, in another study [85], a reduction in this parameter was observed. According to the literature [86], PLA impact strength can be influenced by the degree of crystallinity, the size of the crystals, and the amount of tie molecules between the crystals. Yang et al. [87] obtained a tougher PLA by subjecting it to annealing at 90 °C for 2 h, 8 h, and 24 h. They attribute the improvement in the performance of the annealed PLA to the development of crystallinity and the perfection of the crystallites. Although annealing toughened the PLAT, adding carbon nanotubes had a deleterious effect on the impact strength, evidencing a stress-concentrating effect. The 1 phr MWCNT content in PLAT resulted in an approximately 28.9% reduction in impact strength compared to pure PLAT. However, the PLAT/MWCNT (1 phr) nanocomposite showed 61.4% higher impact strength than unannealed PLA, indicating that it is tougher at room temperature. For the 2 phr, 3 phr, and 4 phr MWCNT contents, the reduction in the impact strength of the PLAT/MWCNT nanocomposites was even more pronounced, with results similar to those of PLA without heat treatment. This behavior can be explained by the higher concentration of MWCNT in the amorphous regions of PLA after annealing, which has accelerated the embrittlement process due to the high rigidity of the carbon nanotubes.

**Figure 12:**
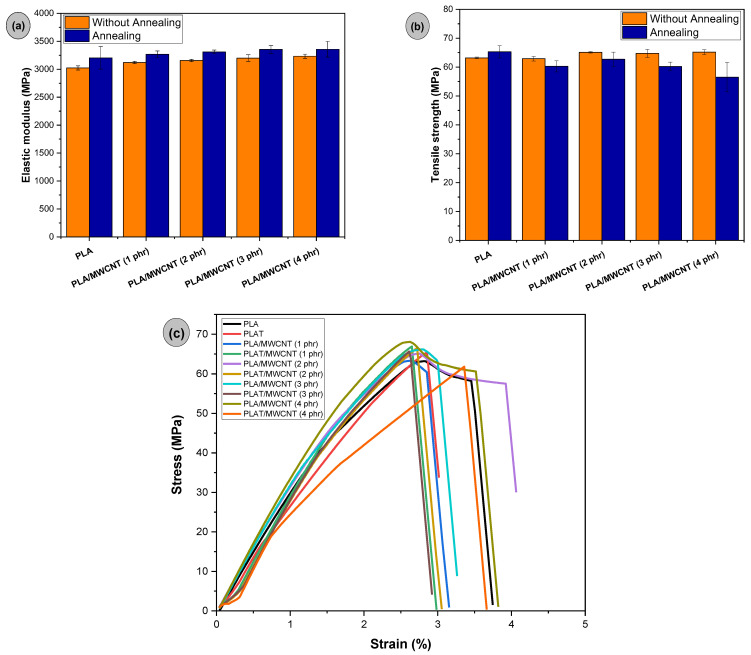
Mechanical properties under tensile for PLA and nanocomposites: (**a**) elastic modulus; (**b**) tensile strength; (**c**) stress vs. strain curves.

Figure 12a,b shows the tensile mechanical properties of PLA and its nanocomposites with varying MWCNT concentrations, as well as the effect of annealing. The elastic modulus of PLA was 3023 MPa, indicating high stiffness at room temperature. As the MWCNT concentration in PLA increased, a slight increase in the elastic modulus was observed, suggesting that the carbon nanotubes restricted the molecular mobility of PLA, leading to higher stiffness. This trend is consistent with the behavior observed in impact strength. For example, the PLA/MWCNT (4 phr) nanocomposite showed an increase of approximately 7% compared to pure PLA. However, overall, the elastic modulus values were similar between the nanocomposites and PLA, considering the experimental error margin. The annealing treatment of PLAT and PLAT/MWCNT nanocomposites increased stiffness, as shown in Figure 12a. PLAT showed an increase in elastic modulus to 3204 MPa, representing a roughly 6% increase compared to non-annealed PLA. The PLA/MWCNT nanocomposites, regardless of MWCNT concentration, exhibited the same trend of increased stiffness after annealing, with values ranging from 3267 to 3358 MPa. The improvement in the elastic modulus of both PLAT and PLAT/MWCNT nanocomposites was attributed to increased crystallinity, as observed in the XRD analysis. During annealing, the molecular chains of PLAT and PLAT/MWCNT nanocomposites underwent reorganization, forming a more ordered structure that facilitated the formation of crystalline regions. These crystalline fractions are more rigid and restrict the rotation of molecular chains, compared to the amorphous structure [88].

Figure 12b shows that the maximum tensile strength of PLA was 63.2 MPa, a value consistent with that reported in the literature [89]. Incorporating 1 phr of MWCNT into PLA did not affect the tensile strength, maintaining the same value. However, when the MWCNT content was increased to 2 to 4 phr, a slight increase in tensile strength was observed, ranging from 64.8 to 65.2 MPa, suggesting a small reinforcing effect. As for the annealing effect, PLAT showed an increase in tensile strength, reaching 65.3 MPa. Crystallinity favored PLAT, improving its tensile strength, probably due to forming crystals with a good amount of interspherulitic tie molecules, contributing to a reinforcing effect. In contrast, PLAT/MWCNT nanocomposites reduced tensile strength compared to non-annealed nanocomposites, with values ranging from 56.5 to 62.7 MPa. As shown in Figure 7, annealing caused the carbon nanotubes to preferentially move to the interspherulitic contours of PLAT. This probably reduced the amount of tie molecules between PLAT crystals, which may have favored premature failure and decreased tensile strength. Figure 12c presents the stress vs. strain curves for PLA and the nanocomposites before and after annealing. In general, the materials presented high maximum stress behavior, but with low strain value. This indicates the typical behavior of a glass polymer at room temperature.

**Figure 13:**
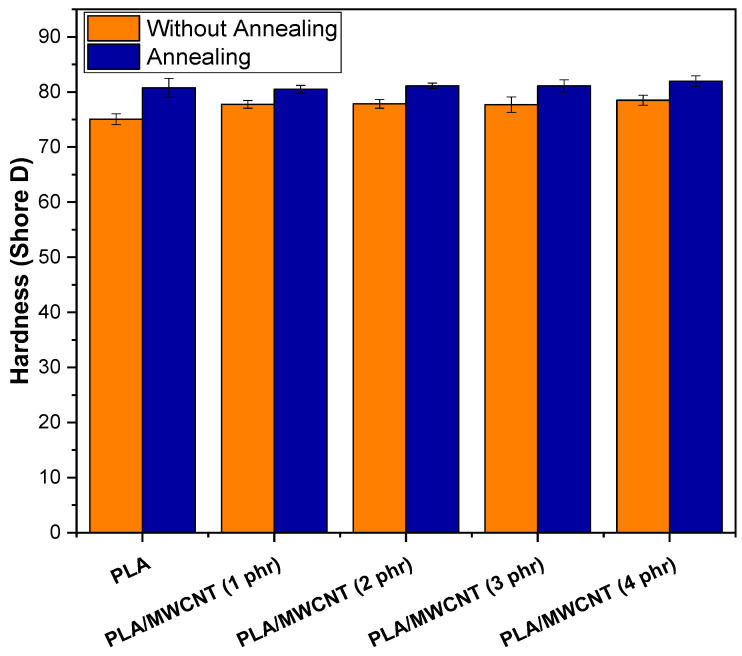
Shore D hardness values for PLA and PLA/MWCNT nanocomposites as a function of MWCNT content and annealing treatment.

Figure 13 shows the behavior of the Shore D hardness of PLA and nanocomposites, as a function of the MWCNT content and the annealing treatment. The hardness of PLA was approximately 75 Shore D, a value consistent with that reported by Norazlina and Kamal [90]. The PLA/MWCNT nanocomposites exhibited Shore D hardness values ranging from 77 to 78.5, indicating greater resistance to surface penetration. This suggests that the carbon nanotubes contributed to the increase in surface hardness, confirming the trend observed in the elastic modulus. The annealing treatment led to an additional increase in Shore D hardness for both PLAT and PLAT/MWCNT nanocomposites, with values ranging from 80 to 82. This enhancement was attributed to the improvement in crystallinity, as observed in the XRD analysis, which provided greater resistance to surface penetration. Furthermore, the results for both PLA and PLA/MWCNT nanocomposites were generally similar, considering the experimental error margin.

### 3.7. Heat Deflection Temperature (HDT)

**Figure 14:**
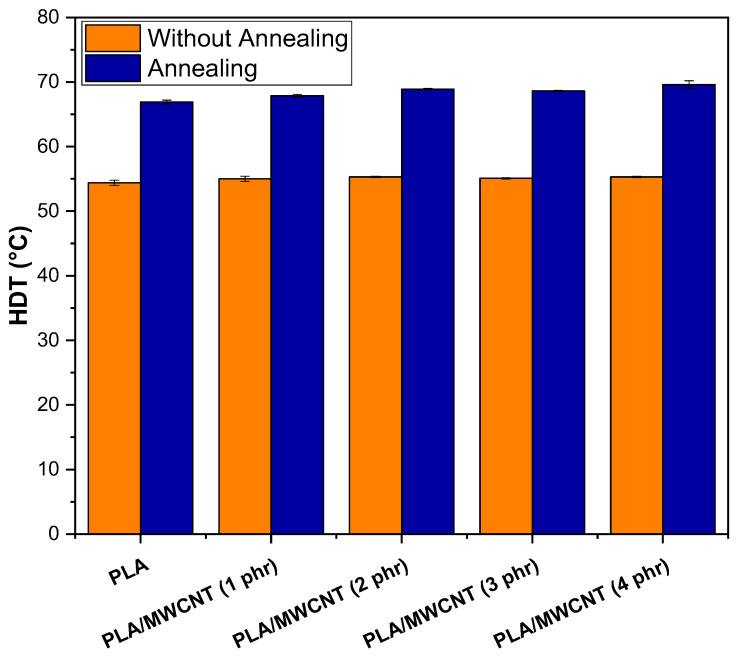
HDT results for PLA and nanocomposites.

HDT is the temperature at which a material begins to deform under load, indicating its thermomechanical strength. Figure 14 shows the HDT behavior of PLA and the nanocomposites before and after annealing.

Figure 14 shows that PLA had an HDT of approximately 54.5 °C, a value consistent with that reported in the literature [91]. The PLA/MWCNT nanocomposites, regardless of the MWCNT concentration, did not significantly improve thermomechanical strength, showing only a slight increase in HDT, ranging from 55 °C to 55.6 °C. In contrast, the HDT values were higher for the PLAT and PLAT/MWCNT nanocomposites after the annealing treatment, this being the most relevant factor for the increase in this property. After annealing, PLAT showed an increase in HDT to 66.9 °C, representing a 22.7% improvement compared to non-annealed PLA. The PLAT/MWCNT nanocomposites followed a similar trend, with an increase of approximately 13.5 °C in HDT compared to their non-annealed nanocomposites, indicating enhanced thermomechanical strength. This increase can be attributed to the enhanced crystallinity, as indicated by the Xc values obtained from the XRD analysis. Additionally, the crystal growth promoted by the annealing process likely contributed to this behavior, as is commonly observed in PLA [92]. The results of this study are consistent with those reported by Pereira and Morales [93].

### 3.8. Differential Scanning Calorimetry (DSC)

**Figure 15:**
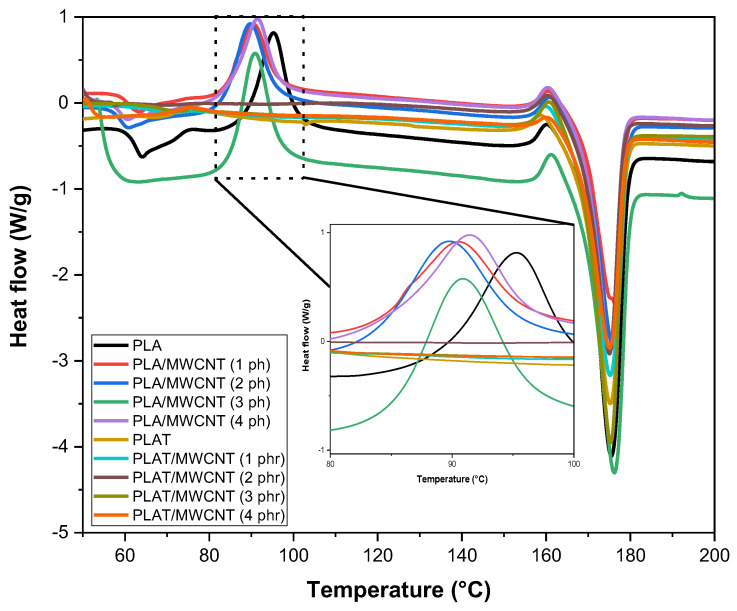
Curves obtained by DSC for PLA and nanocomposites, with and without annealing. Curves relating to the first heating cycle.

Figure 15 shows the DSC behavior of PLA and the nanocomposites before and after annealing. Table 3 summarizes the results of the thermal properties by DSC, especially for glass transition temperature (T_g_), crystalline melting temperature (T_m_), and degree of crystallinity (X_c_).

The pure PLA showed a glass transition (T_g_) around 62.1 °C, a value close to what is reported in the literature [94]. Adding 1 to 4 phr of MWCNT to the PLA matrix shifted the T_g_ to values between 58 and 61 °C. As shown in Table 3, the presence of carbon nanotubes reduced the crystallinity of PLA, which likely contributed to the decrease in the T_g_ of the PLA/MWCNT nanocomposites compared to pure PLA. Generally, lower crystallinity is associated with a reduction in T_g_, as the material becomes more amorphous. When annealing was applied to PLAT, an increase in T_g_ was observed to 65.1 °C, corroborating the results from the literature [62,93]. This indicates a reduction in the amorphous fraction after annealing, which contributed to increased restriction of molecular mobility, consistent with the increase in elastic modulus. In the PLAT/MWCNT nanocomposites, regardless of the MWCNT content, the T_g_ increased to the range of 65–67 °C. For these nanocomposites, the increase in T_g_ is associated with a decrease in the amorphous fraction. That is, with a higher degree of crystallinity, the remaining amorphous chains have less freedom of movement due to the restrictions imposed by the crystalline regions. The crystalline melting temperature (T_m_) of PLA was close to 175.4 °C, a behavior similar to that observed by Zhou et al. [95]. The annealing treatment in PLAT did not significantly alter the T_m_, only maintaining the property value. The PLA/MWCNT and PLAT/MWCNT nanocomposites followed the same trend as the pure matrix, maintaining the Tm between 175–176.5 °C, indicating that there was no abrupt change in the crystalline melting. However, as shown in Figure 15, the cold crystallization temperature disappeared for PLAT and PLAT/MWCNT nanocomposites after the annealing treatment. In this case, the molecules of PLAT were inhibited, preventing sufficient mobility for the chains to rearrange themselves into a crystalline structure during heating, which reflected in the significant increase in the crystallinity degree obtained from the crystalline melting enthalpy (see Equation (2)).

Pure PLA exhibited a low crystallinity degree of 9.7%, indicating the predominance of amorphous material [96]. The PLA/MWCNT nanocomposites showed a crystallinity degree ranging from 8–9%, a value lower than that observed for pure PLA. This behavior suggests that the addition of carbon nanotubes to the PLA matrix did not modify the structural organization, only maintaining the amorphous character. A significant increase in the crystallinity degree was observed for PLAT (50.8%), which is considered indicative of the effectiveness of the annealing treatment, supporting the results from XRD. The application of annealing at 90 °C for 5 h favored the molecular organization of PLAT, resulting in a higher crystalline fraction. For the PLAT/MWCNT nanocomposites, an increase in the crystallinity degree was observed compared to PLAT. This indicates that annealing promoted a nucleating effect of the carbon nanotubes on PLAT. With the reduction of the amorphous fraction of PLAT after annealing, the carbon nanotubes distributed in the PLA chains had a good accommodation, leading to an increased formation of crystal nucleation sites. The maximization of the crystallinity degree was 55.2% for the PLAT/MWCNT (2 phr) nanocomposite, indicating that this was a critical concentration for crystal nucleation. Above 2 phr of MWCNT, the crystallinity degree began to decline again due to the excess of the nanofiller. From a practical standpoint, annealing in PLAT and PLAT/MWCNT nanocomposites was effective in improving the crystallinity degree, which in turn enhanced the thermomechanical stability (HDT), elastic modulus, and Shore D hardness.

### 3.9. Perspectives of PLA/MWCNT Nanocomposites

During the melt processing of PLA with carbon nanotubes (MWCNT), there is a difficulty in achieving good distribution and dispersion, especially due to the high viscosity. This behavior makes processing challenging, requiring specific conditions to prevent the formation of agglomerates. However, the use of a screw in the extruder configured for high shear rates can promote good distribution of carbon nanotubes in PLA, as demonstrated in the PLA/MWCNT (2 phr) nanocomposite. In this case, there was effectiveness in promoting good distribution at low MWCNT concentrations, which also helps reduce the cost of the final product. The mechanical results of the PLA/MWCNT nanocomposites indicated that the performance was comparable to pure PLA, but with a multifunctional behavior by inducing electrical and electromagnetic properties. The PLA/MWCNT (2 phr) nanocomposite showed an electromagnetic shielding result greater than 20 dB (Figure 8), suggesting potential for attenuating electromagnetic radiation. There are several practical applications in real-world scenarios, particularly in areas that require lightweight, sustainable, and efficient materials. For instance, they can be used to produce packaging that protects electronic devices, such as smartphones, tablets, and laptops, from electromagnetic interference. In the automotive sector, which is increasingly dependent on complex electronic systems, the use of such materials can help prevent electromagnetic interference that might affect the functionality of the car’s electronic systems. In wireless communication systems, nanocomposites with radiation-attenuating properties can be used to protect circuits from EMI. This not only helps maintain signal integrity and reduce data loss but also offers a more environmentally friendly solution compared to traditional materials.

The production of PLA/MWCNT nanocomposites in extruders offers several advantages, particularly because it is a continuous process capable of producing large quantities of nanocomposites. Additionally, it is a widely used processing technology in the plastic manufacturing industry due to its ability to produce materials with greater homogeneity, ease of handling, and versatility in adapting operational parameters. An essential requirement is that the screw configuration must be suitable to promote better distribution and dispersion of MWCNT, which enhances electrical, mechanical, and electromagnetic properties. Although PLA is still relatively more expensive than traditional petroleum-based polymers, the growing demand is expected to drive higher production, helping to reduce costs in the future and boost the manufacturing of components.

## 4. Conclusions

PLA nanocomposites reinforced with MWCNT were prepared using the melting mixing technique, and the effects of MWCNT content and annealing on the mechanical, thermomechanical, electrical, and electromagnetic properties were investigated. With the screw profile used in the extruder, the content of 2 phr of MWCNT in the PLA matrix was adequate to produce a nanocomposite with electromagnetic shielding of 20–22 dB, which is suitable for application in technical parts. This meant attenuating around 99% of the electromagnetic wave, with the predominant mechanism being reflection. SEM analysis revealed a good distribution of carbon nanotubes in the PLA matrix, which enhanced both electrical conductivity and the barrier effect against electromagnetic radiation. Additionally, slight improvements in the mechanical and thermomechanical properties of the PLA/MWCNT (2 phr) nanocomposite were observed, indicating a synergistic effect between the evaluated properties. At the same time, the annealing effect on the PLA/MWCNT (2 phr) nanocomposite was particularly evident in improving thermomechanical resistance, as shown by the 13.5 °C average increase in HDT. From a practical standpoint, this resulted in enhanced structural stability at higher temperatures, which could benefit electrical applications while maintaining an attenuation level between 19.7–20 dB. These results suggest the potential for producing PLA-based nanocomposites that are effective for electromagnetic shielding using a processing technique widely employed in the plastics industry.

## Figures and Tables

**Table 1:** Degree of crystallinity, basal spacing (d), and crystal size (L) by XRD for pure PLA and nanocomposites.

Samples	X_c_ (%)	2θ (°)	d (Å) ^1^	L (nm) ^2^
PLAT	46.0	16.98	5.24	20.9
PLAT/MWCNT (1 phr)	53.9	16.90	5.26	25.8
PLAT/MWCNT (2 phr)	63.7	16.88	5.27	26.1
PLAT/MWCNT (3 phr)	51.0	16.98	5.24	29.4
PLAT/MWCNT (4 phr)	47.2	17.06	5.21	26.9

^1^ The interplanar distance (d) was determined by Bragg’s Law (nλ = 2dsin(θ)), using n = 1 and λ = 1.548 Å. ^2^ The crystal size (L) was determined by the Scherrer Equation (L = kλ/βcos θ); k is the Scherrer constant; λ is the wavelength of CuKα radiation; β (rad) is the half-height width of the most intense crystalline peak; and θ (rad) is the Bragg’s angle. The calculation of the crystal size (L) was performed using the Scherrer equation using the DIFFRAC.plus TOPAS software from Bruker (Version 3.0—Bruker AXS).

**Table 2:** Electrical conductivity of PLA and nanocomposites, with and without annealing treatment.

Electrical Conductivity (S/cm)		
Samples	Without Annealing	With Annealing
PLA	(8.13 ± 0.05) × 10^−11^	(8.54 ± 0.04) × 10^−11^
PLA/MWCNT (1 phr)	(2.85 ± 0.02) × 10^−7^	(2.17 ± 0.02) × 10^−7^
PLA/MWCNT (2 phr)	(3.07 ± 0.03) × 10^−6^	(2.65 ± 0.04) × 10^−6^
PLA/MWCNT (3 phr)	(7.46 ± 0.03) × 10^−6^	(8.20 ± 0.07) × 10^−6^
PLA/MWCNT (4 phr)	(7.14 ± 0.06) × 10^−6^	(8.26 ± 0.06) × 10^−6^

**Table 3:** Thermal properties obtained by DSC for pure PLA and nanocomposites.

Samples	T_g_ (°C)	T_m_ (°C)	X_c_ (%)
PLA	62.1	175.4	9.7
PLAT	65.1	175.2	50.8
PLA/MWCNT (1 phr)	60.9	176.2	8.6
PLAT/MWCNT (1 phr)	66.4	175.3	54.5
PLA/MWCNT (2 phr)	58.5	175.8	8.4
PLAT/MWCNT (2 phr)	66.7	175.1	55.2
PLA/MWCNT (3 phr)	58.6	176.2	8.9
PLAT/MWCNT (3 phr)	66.8	175.3	54.1
PLA/MWCNT (4 phr)	58.4	174.9	8.7
PLAT/MWCNT (4 phr)	65.8	175.0	53.6

## Data Availability

The original contributions presented in this study are included in the article. Further inquiries can be directed to the corresponding author.
